# Post-induction Measurable Residual Disease Using Multicolor Flow Cytometry Is Strongly Predictive of Inferior Clinical Outcome in the Real-Life Management of Childhood T-Cell Acute Lymphoblastic Leukemia: A Study of 256 Patients

**DOI:** 10.3389/fonc.2020.00577

**Published:** 2020-04-24

**Authors:** Prashant R. Tembhare, Gaurav Narula, Twinkle Khanka, Sitaram Ghogale, Gaurav Chatterjee, Nikhil V. Patkar, Maya Prasad, Yajamanam Badrinath, Nilesh Deshpande, Pratyusha Gudapati, Shefali Verma, Mahima Sanyal, Florence Kunjachan, Gunit Mangang, Sumeet Gujral, Shripad Banavali, Papagudi G. Subramanian

**Affiliations:** ^1^Hematopathology Laboratory, ACTREC, Tata Memorial Center, Homi Bhabha National Institute, Mumbai, India; ^2^Department of Pediatric Oncology, Tata Memorial Center, Homi Bhabha National Institute, Mumbai, India

**Keywords:** measurable residual disease, hyperleukocytosis, early clearance, T-cell acute lymphoblastic leukemia, multicolor flow cytometry

## Abstract

**Background:** Measurable/minimal residual disease (MRD) status is suggested as a powerful indicator of clinical-outcome in T-cell lymphoblastic leukemia/lymphoma (T-ALL). Contrary to B-cell ALL, reports on T-ALL MRD are limited and mostly based on molecular methods, mainly from developed countries. Multicolor flow cytometry (MFC)-based T-ALL studies are very few. Clinically relevant cut-off levels and ideal time-point for MRD assessment are still inconclusive. In view of lack of T-ALL MRD data from the developing world, we evaluated the prognostic value of MFC-based post-induction (PI)-MRD assessment in T-ALL in the context of standard practice.

**Methods:** We included 256 childhood-T-ALL patients (age < 15 years) treated with a modified-MCP841 protocol, which uses high-dose cytarabine during consolidation, as a part of standard hospital practice. MRD was studied using 10-color 11-antibody MFC with any level of detectable disease being considered positive. Post-induction (PI)-MRD was available in all patients, and post-consolidation (PC) MRD was available mostly in PI-MRD-positive patients (*n* = 88).

**Results:** Three years cumulative-incidence-of-relapse (3years-CIR) in PI-MRD-positive patients was inferior to negative patients (46.3% vs. 18.4%). The median relapse-free-survival (RFS), event-free-survival (EFS) and overall-survival (OS) with hazard ratio (HR) of PI-MRD-positive patients were 21.4 months vs not reached (*p* < 0.0001, HR-4.7), 21.6 months vs. not-reached (*p* = 0.0003, HR-2.01) and 37.3 months vs. not reached (*p* = 0.026, HR-1.64) respectively. RFS, EFS and OS of patients with PI-MRD<0.01% (*n* = 17) were as inferior as PI-MRD ≥ 0.01% in comparison with MRD-negative patients with HR of 4.7 (*p* < 0.0001), 2.45 (*p* = 0.0003), and 2.5 (*p* = 0.029), respectively. Three-years-CIR of patients with hyperleukocytosis (≥100 × 109/L) was also higher (50.5 vs. 27.6%) with inferior RFS, EFS, and OS. Among PI-MRD-positive patients, 3years-CIR, RFS, EFS, and OS of PC-MRD-positive were also inferior to that of negative patients. On multivariate analysis any-level detectable PI-MRD and hyperleukocytosis remained independently associated with inferior RFS, EFS, and OS. A combination of PI-MRD-positive status and hyperleukocytosis identified the patients with the worst clinical outcomes.

**Conclusion:** Detectable PI-MRD using MFC was found to be the strong predictive factor of inferior clinical outcome in T-ALL patients. The combination of PI-MRD status and hyperleukocytosis provides the most influential tool for the management of T-ALL in resource constrained settings from developing world.

## Background

Measurable residual disease (MRD) status has been suggested as the most powerful indicator of clinical outcome in acute leukemia. The clinical value of MRD assessment has been extensively studied in B-cell acute lymphoblastic leukemia (B-ALL) and acute myeloid leukemia (AML) (1–4). Moreover, MRD status has been incorporated in risk-stratification and MRD-guided therapeutic approaches for childhood B-ALL patients (5). However, the data on MRD monitoring in T-cell acute lymphoblastic leukemia (T-ALL) is limited to a few publications (6–9). Reports demonstrating the clinical impact MRD monitoring in real-life management of T-ALL patients from resource-constrained settings like low-and-middle-income group countries (LMIC) are extremely rare. It is particularly important in LMIC as MRD can provide a reliable parameter for the risk-stratification and hence, risk-directed utilization of available resources. In LMIC, patients face challenges like exposure to communicable infections, malnutrition, and lack of adequate supportive care, etc. (10–15). Additionally, due to financial constraints, majority of patients are treated with low-cost therapeutic protocols like MCP-841 (12, 13, 16). Within the background of such challenges, the benefits of MRD monitoring might not reach the expectations as has been reported by developed countries. Hence, studies validating the predictive value of MRD in the standard hospital-based practice in the LMIC are needed.

MRD is reliably monitored using two methods, real-time quantitative polymerase chain reaction-based MRD (PCR-MRD) and multicolor flow cytometry-based MRD (MFC-MRD). PCR-MRD has been known for its challenges like a time-consuming and labor-intensive methodology, extensive standardization and limited applicability (up to 80–85%) etc. (9, 17–19). On the contrary, MFC-MRD has advantages like wider availability, easy processing, and is relatively inexpensive (9, 19, 20). Most importantly, it has a very low turn-around-time making it suitable for quick therapeutic decisions (20). Hence, MFC-MRD can be easily adaptable in the routine clinical practice for T-ALL management, especially in the LMIC with limited resources. Data highlighting the application of MFC-MRD in T-ALL using improved flow cytometric assays with higher sensitivity is limited to few studies. Additionally, reports on other risk-stratification factors like early thymic precursor T-ALL (ETPALL) immunophenotypic subtype and WBC counts from LMIC are scarce. In this report, we stated the prognostic relevance of MRD assessment using ten-color MFC and other risk factors in a large cohort of T-ALL patients from a tertiary cancer reference center in India.

## Patients and Methods

**Figure 1:**
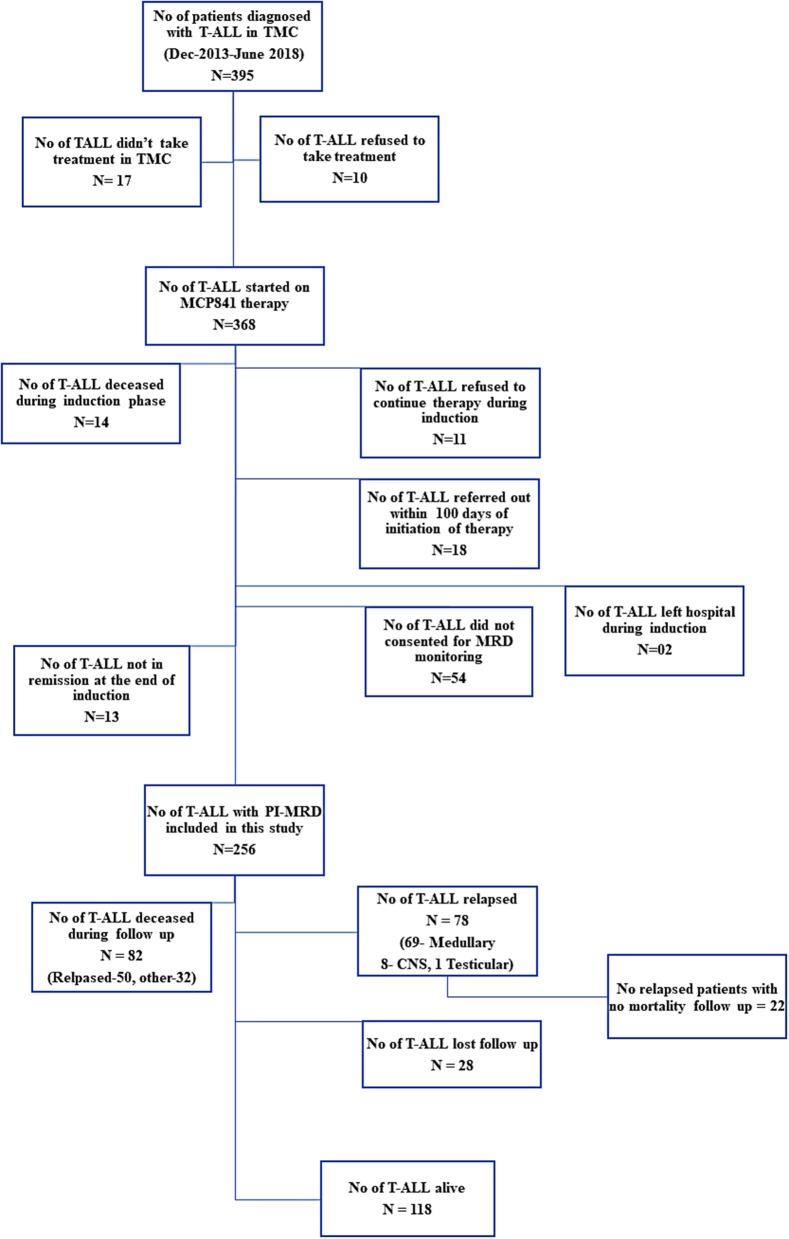
Distribution of patients and the follow-up information available for MRD analysis in the study.

This study included newly diagnosed childhood T-ALL patients (age <15-years) treated with MCP-841 with modification. It was approved by the Hospital Ethics Committee. Patients with partial treatment (who received initial few cycles of chemotherapy in other institute), those who refused to continue treatment in our institute, or with no MRD studies were excluded (Figure 1). Patients who were not in morphological remission and had ≥5% MFC MRD at the end of induction therapy were also exluded from the study. Diagnosis was performed based on the morphologic, cytochemistry (myeloperoxidase) and flow cytometric immunophenotyping (Supplementary Table S1) examinations. Patients were classified into three groups based on the immunophenotype at diagnosis: (1). ETPALL, (2). Near-ETPALL or ETP-like ALL (cases with ETP immunophenotype but normal CD5 expression as described by Children Oncology Group, COG) and (3). Non-ETP T-ALL (21). Patients with Non-ETPALL were further subclassified as per EGIL classification (22).

The MCP-841 protocol was jointly developed by centers in India and the National Cancer Institute (NCI), Bethesda, MD, USA (10, 16). This protocol did not contain high-dose methotrexate, and was designed to be delivered almost entirely in out-patient setting and with minimal supportive care as appropriate for an LMIC. Briefly, the modified MCP-841 protocol included five blocks of intensive chemotherapy followed by maintenance. Patients received a 4-drug induction (vincristine, prednisolone, L-asparaginase, daunomycin), and consolidation with high-dose cytarabine (24 gm/m^2^ divided into three equal legs for children below 3-years, and 16 gm/m^2^ in two equal legs, for 3 or more years age). Post-consolidation (PC) therapy followed the pattern of interim maintenance, then two delayed intensification cycles, and 18 months maintenance. Maintenance included 3-monthly blocks of Vincristine, Daunomycin, and Intrathecal (IT) methotrexate, followed by 1-week blocks of steroids and l-asparaginase. After a gap of 1-week, this was followed by 10-weeks of oral 6-mercaptopurine and weekly oral methotrexate. CNS-directed therapy was consisted of intrathecal methotrexate cycles (induction (I) −3 cycles, I2A −2 cycles, I2 −4 cycles, reinduction −3 cycles, consolidation− 1 cycles and maintenance −6 cycles). Patients with CNS-involvement received additional cranial radiation of 18 Gy. For ETP-ALL, induction therapy was modified to replace prednisolone with dexamethasone, all other components being the same.

## MFC MRD Monitoring

MFC-MRD was performed in BM aspirate samples at an early time-point i.e., at the end of induction (post-induction, PI; day 35–40) and at a later time-point i.e., the post-consolidation (PC-MRD, day 78–83). BM samples for MRD assessment were processed using Euroflow bulk-lysis protocol (20) and an 10-color antibody-panel. In brief, the cell suspension was prepared by bulk erythrocyte lysing with ammonium chloride-based lysing reagent, and the cells were stained with the 11-antibody 10-color MRD panel (Supplementary Table S2). The primary MFC-MRD panel included the antibodies against surface CD3, intracellular CD3, CD4, CD5, CD7, CD8, CD16, CD34, CD38, CD45, and CD56. An additional panel of antibodies against CD1a, CD2, CD11b, CD13, CD33, CD117, and TdT (Supplementary Table S2 in Supplementary Datasheet) was used based on the knowledge of diagnostic immunophenotype only in suspicious samples where primary panel was not found helpful. The cells were acquired on the Navios instrument (Beckman Coulter, Miami, FL, USA). Instrument set-up and daily quality controls were performed as per the manufacturers' recommendations. The limit of detection (LOD) for MRD assay was established at a cluster of 20 events in 1.5 million cells (i.e., 0.0013%) and lower limit of quantitation (LOQ) at a cluster of 50 events 1.5 million cells (i.e., 0.003%). Median number of events acquired was 1,538,935 (range, 823,148–5,632,547 events) and we could acquire ≥1.5 million cells in 63% of MRD samples. All samples had a detection sensitivity of ≤0.00625%. Flow cytometry data analysis was performed using Kaluza-software version 1.3 (Beckman Coulter, Miami, USA), as demonstrated in Supplementary Figures S1, S2.

## Statistical Analysis

The association between baseline-characteristics and early clearance of (PI-MRD) was evaluated using Fisher's exact test and between age and incidence of ETP-subtype using logistic regression. Relapse-free survival (RFS) was calculated from the date of the post-induction BM examination until the date of relapse of the disease in bone marrow or peripheral blood (medullary relapse). Overall survival (OS) was calculated from the date of prephase initiation until the date of death. Event-free survival (EFS) was calculated from the date of prephase initiation until the date of any event that included medullary relapse or isolated CNS or testicular relapse or relapse-free death. The association of various factors such as age, total WBC count at diagnosis, immunophenotypic sub-type (ETPALL/Near-ETP vs. Non-ETPALL), PI-MRD status and PC-MRD status with RFS, OS, and EFS was studied by using Kaplan–Meier method and cumulative incidence of relapse (CIR) were determined. Multivariate analysis was performed using a Cox proportional hazards model. A *p* < 0.05 was considered statistically significant. Statistical analysis of the data was performed using MedCalc Statistical Software version 14.8.1® (MedCalc Software, Ostend, Belgium), and Stata version13® (StataCorp LP, Tx, USA).

## Results

### Patients and Samples

Between December 2013 and June 2018, 395 children were diagnosed with T-ALL. Of them, 368 were started on the modified-MCP841 protocol. After applying exclusion criteria (which included: incomplete induction therapy, referral to other hospital, induction deaths, unavailability of MRD samples, and not-in-remission status at the end of induction therapy), 256 patients were included in this study (Figure 1). The detailed hierarchical distribution of patients is given in Figure 1, and their characteristics are described in Table 1. Median age (9.5 years) and male-to-female ratio (3.9) in T-ALL were higher than B-ALL patients (published elsewhere) (20). Median WBC count at diagnosis was 88.4 × 10^9^/L and hyperleukocytosis (defined as WBC at diagnosis ≥100 × 10^9^/L) was found in 46.5% of cases. Patients were immunophenotypically subclassified as ETPALL (34, 13.3%), Near-ETPALL (10, 3.9%) and Non-ETPALL (214, 82.8%). The incidence of ETPALL was found to be higher with increment in the age of the patients (*p* = 0.01) with OR of 1.05 (95 CI, 0.97–1.18). Notably, there was no patient with ETPALL below the age of 6 years and the median age for ETPALL was 10.4 years (range, 6–14.6 years).

**Table 1:** Characteristics of T-cell Lymphoblastic Leukemia patients (*n* = 269).

**Characteristics**	**Values**
**Age (in years)**	
•Median	9.5 years
•Range	9 months−15 years
Males	204 (79.69%)
Females	52 (20.31%)
Male: Female ratio	3.92
**WBC counts**	
•Median	88.41 × 103/μL
•Range	0.83–849.92 × 103/μL
•Patients with ≥100 X 103/μL	46.50%
**Blast % at diagnosis**	
•Median	81.50%
•Range	25–98.50%
**CNS Status**	
•CNS-I	229 (89.45%)
•CNS-II	002 (00.78%)
•CNS-III	025 (09.77%)
**Extramedullary disease**	
•Testicular involvement	07 (02.73%)
•Mediastinal disease	73 (28.52%)
•Bulky or generalized LN enlargement	18 (07.03%)
**Immunophenotypic category**	
•ETP-ALL (*n* = 34)	13.28%
•Near ETP-ALL (*n* = 10)	3.91%
•Non-ETPALL (*n* = 212)	82.81%
°EGIL-I	03.31%
°EGIL-II	19.19.6%
°EGIL-III	63.10%
°EGIL-IV	14.10%
**Morphological (bone marrow)**	
remission status^*^	
•In remission (*n* = 247)	96.48%
•Not in remission (*n* = 9)	03.52%
**MRD positive status**	
•PI (*n* = 256)	43.75%
PI-MRD levels: Median (Range)	0.22% (0.0007–3.31%)
PI-MRD >0.01%	36.69%
•PC (*n* = 97)	33%
PC-MRD levels: Median (Range)	0.025% (0.0008–4.6%)
PC-MRD >0.01%	26.78%

* *Patients (n = 13) who were not in morphological remission as well as had ≥5% MFC-MRD were excluded initially*.

*ETP, early thymic precursor; LN, lymph node; PI, post-induction; PC-post consolidation*.

### MRD Results

PI-MRD was measurable in 112/256 (43.8%) samples with a median MRD level of 0.22% and range of 0.0007 to 3.3% (MRD >0.01% in 36.7%). PC-MRD was available in 97/256 patients, in which 88 patients were PI-MRD positive. PC-MRD was measurable in 32/97 (33%) with a median MRD level of 0.025% and range from 0.0008 to 4.6% (MRD >0.01% in 26.8% PC samples). None of the nine patients who had undetectable MRD at post-induction had detectable MRD at post-consolidation monitoring. Thus, in 112 PI-MRD positive patients, PC-MRD was available in 88 patients with positive MRD in 32/88 (36.4%).

### Association Between Patients' Characteristics and MRD Status

Of the baseline characteristics studied, ETP-subtype (*n* = 34) had higher PI-MRD positivity rate (56.2 vs. 43.7%; *p* = 0.009). It was even more significant on combining ETP and near-ETP (total *n* = 44) versus (vs.) non-ETP ALL (68 vs. 43.7%; *p* = 0.0001). There was no association between ETPALL subtype (*n* = 14 in 88) and PC-MRD status (*p* = 0.34).

### Follow Up and Outcome

This study included 256 childhood T-ALL with a median follow-up of 26.8 months (range, 1.1–68 months). The follow-up information for relapse or mortality was missing in 28 (10.9%) patients (median follow-up, 8.8-months; range, 3–25.6 months) as they were referred to a local physician for further treatment due to family constraints. Of 256, 69 (26.95%) developed medullary relapse on follow-up with a median duration to relapse of 10.9 months (range, 1.4–49.7 months). Fifteen patients developed CNS-relapse of whom eight had isolated CNS-relapse. One patient developed isolated testicular-relapse. Sixty-five patients with medullary relapse were either referred to local hospitals for palliative care or hematopoietic stem cell transplant (HSCT) in other centers and so, mortality status was not available in 22/65 (33.8%) patients. Total eighty-two (32%) patients died during treatment and follow up. Of them, 50 (61%) were due to relapse (46 medullary & 4 CNS relapse), and remaining deaths were due to other causes that included 8 (9.8%) fungal sepsis or pneumonia, 5 (6.1%) viral encephalitis, 4 (4.9%) drug toxicity, 4 (4.9%) septicemia, 1 (1.2%) multidrug resistance tuberculosis, 5 (6.1%) viral infections like Chickenpox virus, Herpes Simplex Virus Influenza, and Hepatitis-C-Virus, etc. In 5 (6.1%) patients cause of death was not known.

### MRD Status and Outcome

**Figure 2:**
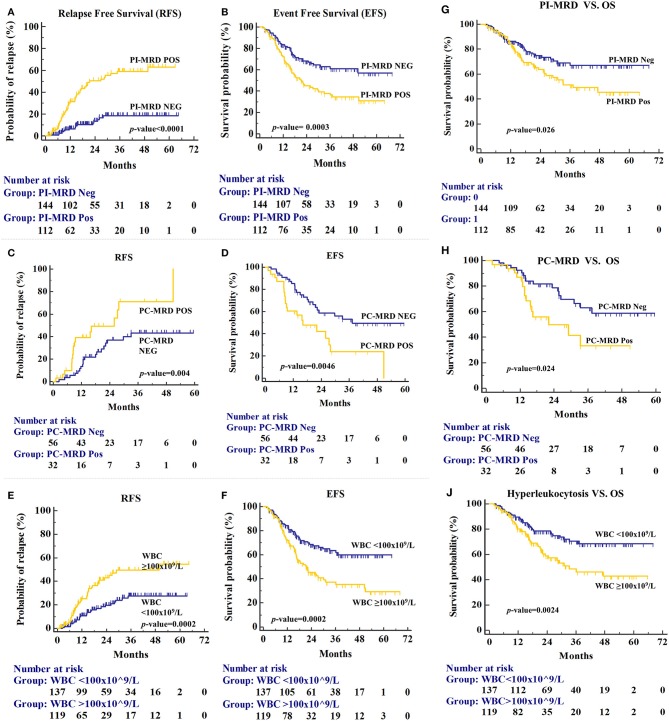
Relapse-free survival (RFS) for patients stratified by PI-MRD status **(A)**, PC-MRD status **(C)**, and hyperleukocytosis status **(E)**. Similarly, Event-free survival (EFS) for PI-MRD status **(B)**, PC-MRD status **(D)**, and hyperleukocytosis status **(F)** (Kaplan–Meier analysis). Overall Survival (OS) for PI-MRD status **(G)**, PC-MRD status **(H)**, and hyperleukocytosis status **(J)** (Kaplan–Meier analysis).

Prognostic values of MRD status and other risk factors are given in Table 2. Of 112 PI-MRD positive patients, 52 (46.43%) developed medullary-relapse while in 144 PI-MRD negative patients, only 17 (11.81%) developed medullary-relapse. PI-MRD positive patients showed a higher risk of relapse with a median RFS of 21.4 months (95% CI, 28.47–39.17 months) versus not-reached in negative patients (Figure 2A) with HR for relapse of 4.714 (95% CI, 2.90–7.63; *p* < 0.0001). Three-years CIR in PI-MRD positive patients was 46.3% while in negative patients was 18.4%. Similarly, the incidence of any event was also higher in PI-MRD positive when compared to negative patients (56.25% versus 31.94%; *p* = 0.0003). PI-MRD positive status was strongly associated with shorter EFS with a median of 21.6 months vs. not-reached in negative patients (Figure 2B). HR of any event for PI-MRD positive patients was 2.01 (95% CI, 1.34–2.41; *p* = 0.0003). The OS was reduced in PI-MRD positive when compared to negative patients (61.6 vs. 69.1%; *p* = 0.026). PI-MRD positive status was strongly associated with shorter OS with median OS of 37.3 months (95% CI 35.27–46.16 months) vs. not-reached in PI-MRD negative patients (Figure 2G) and HR was 1.64 (95% CI 1.05–2.56).

**Table 2:** Univariate analysis of MRD and other covariates with RFS, EFS and OS as the outcome.

	**RFS**			**EFS**			**OS**		
		**95% CI**	***p*-value**		**95% CI**	***p*-value**		**95% CI**	***p*-value**
**Age** **>10 years (*****n*** **=** **256)**									
HR	1.10	0.69–1.78	0.68	0.89	0.61–1.3	0.55	0.87	0.56–1.36	0.56
RR	1.14	0.76–1.70	0.53	0.92	0.68–1.24	0.58	0.95	0.65–1.38	0.78
OR	1.19	0.68–2.08	0.53	0.87	0.52–1.44	0.58	0.93	0.54–1.59	0.78
**Hyperleukocytosis (*****n*** **=** **256)**									
HR	2.42	1.5–3.9	0.0002	2.01	1.38–2.95	0.0003	1.96	1.26–3.07	0.0024
RR	1.9	1.25–2.9	0.0027	1.74	1.29–2.34	0.0003	1.60	1.1–2.33	0.013
OR	2.42	1.37–4.26	0.0023	2.63	1.58–4.38	0.0002	1.99	1.16–3.40	0.012
**Mediastinal mass (*****n*** **=** **256)**									
HR	0.79	0.47–1.33	0.40	1.12	0.70–1.64	0.73	1.17	0.72–1.91	0.50
RR	0.82	0.51–1.32	0.41	1.05	0.77–1.44	0.73	1.16	0.78–1.71	0.45
OR	0.76	0.41–1.44	0.40	1.10	0.63–1.90	0.73	1.24	0.70–2.22	0.46
**ETP–subtype (*****n*** **=** **256)**									
HR	0.96	0.48–1.92	0.90	0.80	0.46–1.39	0.46	1.03	0.54–1.96	0.90
RR	0.98	0.54–1.79	0.94	1.22	0.502–1.32	0.41	1.16	0.79–1.72	0.45
OR	0.97	0.43–.220	0.94	1.40	0.34–1.52	0.38	1.24	0.7–2.2	0.45
**PIMRD** **+** **status (*****n*** **=** **256)**									
HR	4.71	2.90–7.63	<0.0001	2.01	1.40–3.00	0.0003	1.64	1.05–2.56	0.026
RR	3.93	2.41–6.41	<0.0001	1.80	1.34–2.41	0.0001	1.53	1.06–2.22	0.022
OR	6.47	3.46–12.13	<0.0001	2.82	1.69–4.72	0.0001	1.87	1.09–3.19	0.022
**PCMRD** **+** **status in PI–MRD+** **cases (*****n*** **=** **88)**									
HR	2.50	1.19–5.25	0.004	2.29	1.17–4.48	0.0048	2.19	1.01–4.71	0.023
RR	1.50	0.96–2.6	0.072	1.50	1.01–2.30	0.046	1.64	0.94–2.86	0.080
OR	2.21	0.91–5.36	0.080	2.39	0.98–5.83	0.05	2.22	0.90–5.45	0.086

*CI, confidence interval; EFS, event-free survival; HR, hazard ratio, OR, Odd's ratio; OS, overall survival, RFS, relapse-free survival; RR, relative risk*.

**Figure 3:**
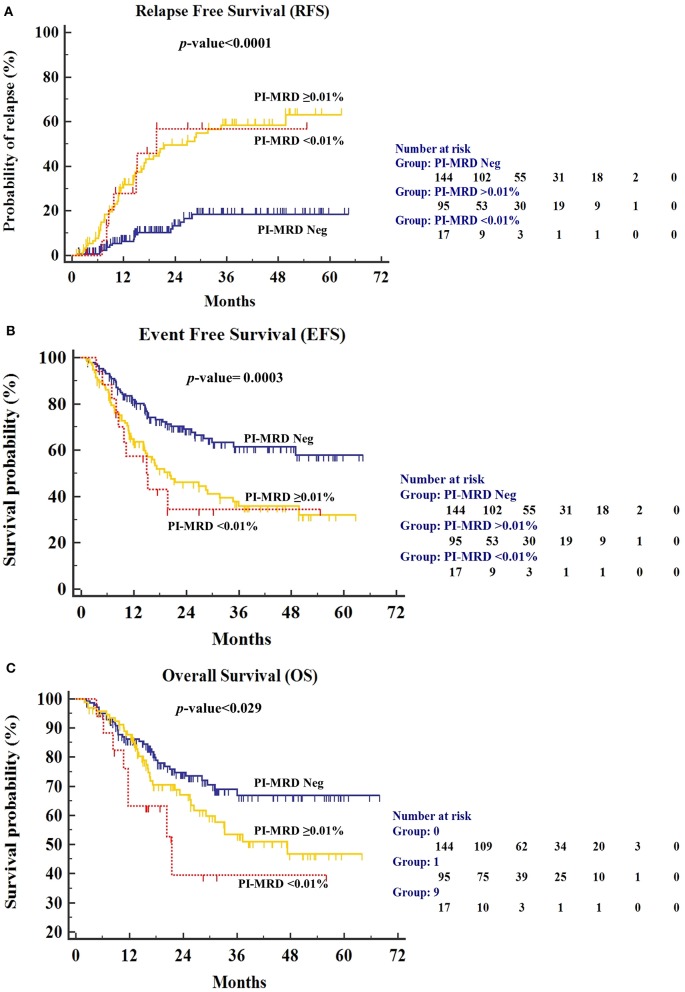
**(A)** Relapse-free survival (RFS), **(B)** Event-free survival (EFS), and **(C)** Overall Survival (OS) for patients stratified by PI-MRD levels being grouped into PI-MRD ≥0.01%, PI-MRD ≤0.01% and PI-MRD not measurable (Kaplan–Meier analysis).

We have also studied the outcome difference between patients with PI-MRD ≥0.01% (*n* = 95), PI-MRD <0.01% (*n* = 17) and PI-MRD negative status (*n* = 144). Although, the group of patients with PI-MRD levels <0.01% was small, the results were similar to patients with PI-MRD ≥0.01% levels (median-RFS 19.7 months, 95% CI, 18.9–43.5 months; median-EFS 15.2 months, 95% CI, 15–37.2 months; and median-OS 21.5 months, 95% CI, 18.3–43.1 months) with HR for relapse, shorter EFS and OS of 4.7 (95% CI, 1.62–13.77; *p* < 0.001), 2.45 (95% CI, 1.06–5.7; *p* = 0.0003), and 2.5 (95% CI, 0.91–6.9; *p* = 0.029), respectively (Figure 3).

Further, we evaluated the significance of PI-MRD negative status (*n* = 144) against PI-MRD positive but PC-MRD negative status (PIMRD+/PCMRD-; *n* = 56). Three-years CIR in PIMRD+/PCMRD- patients was 46.1% while in negative patients was 18.4% with higher risk of relapse (RFS, median- 39.7 months (95% CI, 33.5–46.4 months) and HR for relapse 2.83 (95% CI, 1.39–5.83; *p* = 0.0011) (Supplementary Figure S3). However, although there was a trend but the association between PIMRD+/PCMRD- and EFS as well as OS was not statistically significant (*p* > 0.05). This finding further confirms that even MRD status at the end of induction is a clinically relevant factor for the prediction of risk of relapse even in the absence of detectable PC-MRD.

PC MRD was available predominantly in PI-MRD positive patients and the prognostic significance of PC-MRD was evaluated in these patients (*n* = 88). The PC-MRD positive status in PI-MRD positive patients was also significantly associated with shorter RFS with median RFS of 25.9 months vs. not-reached in PCMRD negative patients (Figure 2C) and HR of 2.5 (95% CI, 1.19–5.25; p = 0.004). Three-years CIR was 71% in PC-MRD positive patients, while 43.1% in negative patients. The time-to-relapse as well as for any event and OS was significantly shorter in PC-MRD positive compared to negative patients (median time-to-relapse, 25.9 months vs. not reached; *p* = 0.004 and median time-to-event, 16.1 vs. 37.3 months; *p* = 0.0046 and median OS, 22.1 months vs. not-reached; *p* = 0.024) (Figures 2D,H).

On multivariate analysis, PI-MRD was found as the most significant and independent high-risk factor for shorter RFS, EFS, and reduced OS with HR, 5.04 (95% CI, 2.89–8.81; *p* < 0.0001), 2.22 (95% CI, 1.49–3.29; *p* = 0.0001) and 1,74 (95% CI, 1.26–3.10 *p* = 0.018), respectively (Table 3).

**Table 3:** Cox proportional-hazards regression analysis with RFS, EFS, and OS as the outcome (*n* = 256).

**Factors**	**HR**	**Std Err**.	***P*-value**	**95% CI**
**Short Relapse Free Survival (RFS)**				
Age > 10 years	1.20	0.258	0.4835	0.72–1.98
Hyperleukocytosis	2.49	0.258	0.0004	1.50–4.11
Mediastinal mass	1.10	0.286	0.7181	0.63–1.93
ETP-subtype	0.65	0.376	0.2493	0.31–1.35
PIMRD positive status	5.04	0.286	<0.0001	2.89–8.81
**Short Event Free Survival (EFS)**				
Age > 10 years	0.94	0.20	0.7756	0.63–1.41
Hyperleukocytosis	2.04	0.20	0.0004	1.37–3.03
Mediastinal mass	1.32	0.21	0.2037	0.86–2.01
ETP-subtype	0.73	0.32	0.3235	0.39–1.36
PIMRD positive status	2.22	0.20	0.0001	1.49–3.29
**Short Overall Survival (OS)**				
Age > 10 years	0.86	0.24	0.5404	0.53–1.31
Hyperleukocytosis	1.90	0.23	0.0031	1.20–2.87
Mediastinal mass	1.40	0.24	0.1738	0.89–2.23
ETP-subtype	1.02	0.34	0.9477	0.45–1.71
PIMRD positive status	1.74	0.24	0.0184	1.26–3.10

### Other Characteristics and Outcome

Of the baseline characteristics studied, only hyperleukocytosis was found as a high-risk factor for relapse (36.13 vs. 19%; *p* = 0.0002), shorter EFS (21.6 months vs. not reached; *p* = 0.0002) and reduced OS (33.3 months vs. not-reached; *p* = 0.0024) (Figures 2E,F,J). Three-years CIR in patients with hyperleukocytosis was also higher than patients with lower WBC counts (50.5 vs. 27.6%). HR of hyperleukocytosis for relapse, shorter EFS & OS were respectively 2.42 (*p* = 0.0002), 2.02 (*p* = 0.0002), and 1.96 (*p* = 0.0024) (Table 2). Hyperleukocytosis was also found to be an independent high-risk factor for relapse, EFS and OS on multivariate analysis with HR for relapse of 2.49 (*p* = 0.0004), any event of 2.04 (*p* = 0.0004) and reduced OS of 1.90 (*p* = 0.0031) respectively (Table 3).

### Clinical Relevance of Combination of PI-MRD Status and Total WBC Count

**Figure 4:**
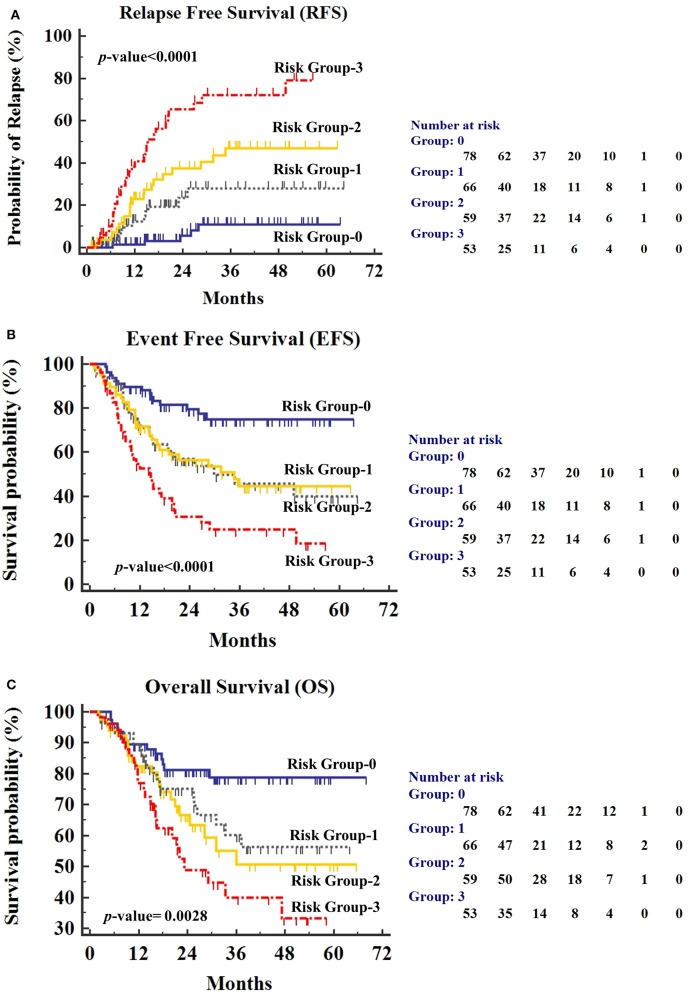
**(A)** Relapse-free survival (RFS), **(B)** Event-free survival (EFS), and **(C)** Overall Survival (OS) for patients stratified by PI-MRD status and hyperleukocytosis status being grouped into “Group-0” (PI-MRD negative with no hyperleukocytosis), “Group-1” (PI-MRD negative but with hyperleukocytosis), “Group-2” (PI-MRD positive without hyperleukocytosis), and “Group-3” (PI-MRD positive with hyperleukocytosis) (Kaplan–Meier analysis).

Hyperleukocytosis and PI-MRD positive status were found to be independently associated with shorter RFS, EFS, and OS. Using these two factors, we divided the patients (*n* = 256) into four risk groups; “group-0” patients with low WBC at diagnosis (<100 × 109/L) and PI-MRD negative status; “group-1” patients with hyperleukocytosis and PI-MRD negative status; “group-2” patients with low WBC and PI-MRD positive status; finally, “group-3” patients with hyperleukocytosis and PI-MRD positive status. Median RFS of these four risk groups were: group-3, 15.2 months (95% CI, 11.2–20.5) vs. not-reached for group-2, 1, and 0. The risk of relapse for group-0 vs. group-1 vs. group-2 vs. group-3 were 6.4% vs. 18.2% vs. 35.6% vs. 58.5% (*p* < 0.0001) (Figure 4A). Three-years CIR for group-0, 1, 2, 3 were respectively 11%, 27.9%, 40.5%, and 72.1%. Median EFS of these groups were: group-3, 14.7 months vs. group-2, 34.7 months vs. group-1, 29.9 months vs. group-0, did not reach (*p* < 0.0001) (Figure 4B) and median OS were: group-3, 23.3 months vs. group-2,1, and 0 did not reach (*p* < 0.0001) (Figure 4C). HR for relapse, any event, and reduced OS in all four groups are stated in Supplementary Table S3. These results have demonstrated that patients with low WBC count at diagnosis and PI-MRD negative status have a very low risk of relapse and longer EFS and OS while patients with hyperleukocytosis and PI-MRD positivity have a very high risk of relapse and shorter EFS and OS.

We have also investigated the association of hyperleukocytosis in patients with PI-MRD negative status (*n* = 48) with non-relapse deaths (*n* = 22). It showed a trend towards the association that did not reach statistical significance (*p* = 0.13).

## Discussion

MRD-derived prognostic information in T-ALL can influence the choice of treatment options in routine practice (7, 23). In standard practice, the rapid availability of MRD status using MFC can be very helpful in real-time therapeutic decision making. It is particularly necessary for resource-limited settings where host factors like malnutrition, drug toxicities, higher rates of infections, exposure to communicable diseases, poor financial conditions, limited therapeutic options, also play a role in decision making along with MRD status (11, 13–15, 24). Practical benefits of MRD monitoring in the background of these challenges in resources constrained settings are unknown. Herein, we have evaluated the predictive value of MRD monitoring in the standard management of childhood T-ALL treated with a low-cost MCP-841-protocol at a reference cancer center from India. Despite the challenges of LMIC, our data demonstrated that patients who failed to early-clearance of MRD had a significantly higher risk of relapse and inferior clinical outcomes (EFS and OS). Results of this study established the independent prognostic value of MFC-based PI-MRD status in standard practice as reported in earlier studies (6, 8, 9, 17, 21, 25, 26).

In T-ALL, studies have highlighted the prognostic impact of MRD-status assessment at two-time points i.e., at the end of induction (PI-MRD) and consolidation (PC-MRD). In 2011, AIEOP-BFM-ALL 2000 study demonstrated that PC-MRD was a better predictor relapse than PI-MRD. However, the PI-MRD positivity rate was very high in this study with only 16% of patients with negative MRD-status (26). On the other hand, recent studies by COG, NOPHO group, and others have shown PI-MRD as the most relevant predictor of relapse (9, 21, 27, 28). In pediatric patients, the BM procedure needs hospitalization and is performed under anesthesia, which adds to the burden of hospital resources and patient's financial arrangements. In the standard practice (especially in LMIC countries) it may not be feasible to perform MRD at multiple time points. Therefore, it is crucial to select the time-points for the MRD assessment carefully. In this study, PI-MRD was evaluated in all patients but PC-MRD was performed mainly in PI-MRD positive patients. The results have shown that PI-MRD positive status was a superior indicator of short RFS, EFS and reduced OS which is in line with the results of other studies (6, 9, 21, 27). We further evaluated the clinical value of only PI-MRD positive but PC-MRD negative status. Three-years CIR in PIMRD+/PCMRD- patients was higher compared to PI-MRD negative patients (46.1 vs. 18.4%) with HR for relapse 2.83. However, the association between PIMRD+/PCMRD- status and EFS or OS did not reach the statistical significance, which may be because of the fact that the mortalities were also contributed by infectious causes. This finding indicated that an early achievement of MRD negative status is clinically relevant for the longer RFS.

Nevertheless, our data further showed that the persistence of detectable PC-MRD was also associated with the worse clinical outcome (3-years CIR, 71% & HR of relapse, 2.56) suggesting the additional clinical value of PC-MRD monitoring in PI-MRD positive patients. It is very unlikely to get measurable PC-MRD if PI-MRD is negative. Hence, in resource-limited settings, this approach (evaluating PI-MRD status in all and limiting PC-MRD evaluation in only PI-MRD-positive patients) might work more effectively.

Published reports have highlighted different cut-off levels for the prediction of clinical outcome in childhood T-ALL patients. Earlier AIEOP-BFM group has demonstrated an MRD level of ≥0.1% (26), and later on, using the same protocol Paganin et al. have proposed the MRD ≥ 0.05% as significant (27). Recent studies by COG, NOPHO, and French Acute Lymphoblastic Leukemia Study Group (FRALLE), etc. have consistently demonstrated that MRD ≥ 0.01% was clinically significant (9, 21, 28). In agreement with these studies, our data also shows that patients with PI-MRD ≥0.01% had a poor clinical outcome. Moreover, in this study, a small group of patients (*n* = 17) with MRD levels <0.01% was also found to be associated with inferior clinical outcomes. This finding can be attributable to the underestimation of MRD levels (lower than the true value) due to issues commonly seen in standard practice such as hemodilution, scarcity of material, etc.

Traditionally, hyperleukocytosis has been considered as a poor prognostic indicator in ALL (28–30). We also evaluated the value of WBC count in the risk-stratification scheme of our patients. Median WBC (88.4 × 10^9^/L) and proportion of patients with hyperleukocytosis (46.5%) in our study were comparable to that of NOPHO group (29). They reported hyperleukocytosis as a high-risk factor for 5-years EFS in B-ALL but not in T-ALL; contrary to that we have found hyperleukocytosis as an independent poor prognostic factor for relapse (3-years CIR, 50.5%; HR for relapse, 2.4) as well as EFS and OS. A study by Petit et al. has also shown similar results suggesting a poor prognostic value of higher (≥200 × 10^9^/L) WBC counts in T-ALL (28).

Notably, studies emphasizing the combined impact of PI-MRD and hyperleukocytosis in the management of childhood T-ALL are extremely rare. In the present study, we combined the results of PI-MRD status and hyperleukocytosis and formed four risk groups (i.e., group-0, 1, 2, & 3). Of them, group-3 (PI-MRD positive with hyperleukocytosis) patients had a very high risk of relapse and shorter EFS (14.7 months) as well as reduced OS (23.3 months) while group-0 (PI-MRD negative with no hyperleukocytosis) had the better clinical outcome (3-years CIR, 72.1 vs. 11%). These findings highlighted the predictive impact of the combination of PI-MRD status and WBC count at diagnosis for the risk-stratification and thus, provided the relevant basis for making treatment decisions in the management of T-ALL in resource-constrained settings.

Other baseline characteristics, including ETP-immunophenotype, did not show an independent association with the inferior outcome, which is in line with the published reports by the BFM group and others (21, 31). The observed frequency of ETPALL in our cohort (14.1%) was within the range as reported in published studies (21, 32–34). We observed higher frequency of measurable PI-MRD in patients with ETPALL subtype (*n* = 34) compared to non-ETPALL (56.2 vs. 43.7%; *p* = 0.009) which increased further on combining ETP and near-ETP (total *n* = 44) versus non-ETP ALL subtype (68 vs. 43.7%; *p* = 0.0001). These results line-up with previous reports emphasizing the fact that ETPALL and near-ETPALL have a different biology and inferior disease clearance rate (21, 27, 32, 33). We did not find any association between ETPALL and PC-MRD status which can be explained by the fact that in our protocol, consolidation with high-dose cytarabine may have also been beneficial for ETPALL patients.

Our protocol also would have had an influencing role in the outcome, and our data must be interpreted in this context. Overall outcomes of T-ALL were inferior to those reported in developed countries. To some extent, this may be due to a high number of patients with late presentation and higher tumor burden. Of concern also was non-relapse mortality (35/86; 40.7%), mostly due to infections and communicable diseases, which could be detrimental to the predictive value of MRD. However, despite these limitations, our results showed highly significant differences between the MRD-positive vs. MRD negative patients.

Thus, in this study, we report the prognostic impact of MFC-based MRD status assessment on the management of childhood T-ALL in clinical practice from a reference cancer center. We have demonstrated that irrespective of the difficulties and challenges of the developing world, PI-MRD is the most relevant prognostic factor in T-ALL. Our data has also highlighted the independent prognostic value of high WBC count at diagnosis. Finally, we have shown that the combination of PI-MRD positive status and hyperleukocytosis provides a very useful prognostication approach in the routine management of T-ALL.

## Declaration of Earlier Presentations In Meetings

- A part of this study was presented in the 22nd Congress of European Hematology Association Madrid, Spain, June 22–25, 2017. EHA abstract is published in “Haematologica | 2017; 102(s2) | 345” Abstract No E837.
- A part of this study has been also submitted in the ASH 2019. The abstract has been published as “Tembhare P, Narula G, Chatterjee G, et al.: Flow-Cytometry Based Detection of Any Minimal Residual Disease (FC-MRD) in Children with T-Acute Lymphoblastic Leukemias (T-ALL) Is a Powerful Indicator of Outcome. Blood 134:2585-2585, 2019.”

## Data Availability Statement

All datasets generated for this study are included in the article/Supplementary Material.

## Ethics Statement

The studies involving human participants were reviewed and approved by Institutional Ethical Committee-2, Tata Memorial Center, Mumbai. Written informed consent from the participants' legal guardian/next of kin was not required to participate in this study in accordance with the national legislation and the institutional requirements.

## Author Contributions

PT designed and performed the study, performed the data analysis, interpreted the data, statistical analysis, and wrote the paper. PS and GC performed the study, interpreted the data, and wrote the paper. NP, SGu, and PG performed the study and interpreted the data. SGh, YB, and ND performed the quality control of study and processed the samples. FK, TK, SV, GM, and MS performed the study and collected the data. GN, MP, and SB recruited patients, performed clinical analyses, and wrote the paper. All authors contributed to the manuscript writing and approved the final version of the manuscript.

## Conflict of Interest

The authors declare that the research was conducted in the absence of any commercial or financial relationships that could be construed as a potential conflict of interest.
