# Supplementary material for: Post-induction Measurable Residual Disease Using Multicolor Flow Cytometry Is Strongly Predictive of Inferior Clinical Outcome in the Real-Life Management of Childhood T-Cell Acute Lymphoblastic Leukemia: A Study of 256 Patients
Source: Front Oncol. 2020 Apr 24;10:577. doi: 10.3389/fonc.2020.00577 (PMC7193086; doi:10.3389/fonc.2020.00577)
Supplement: Supplementary file 1 [file Table_1.DOCX]

**Supplementary datasheet**

**Title:** **Post-induction measurable residual disease using multicolor flow cytometry is strongly predictive of inferior clinical outcome in the real-life management of T-cell acute lymphoblastic leukemia: A study of 256 patients.**

**Supplementary Table S1. 10-color acute leukemia antibody panel.**

| Fluorochromes | BV510 | BV421 | FITC | PE | ECD/ PE-CF594 | PerCP-Cy5.5/  PC5.5 | PC7 | APC | [APC AF700](mailto:APC_Af@700) | [APC AF750](mailto:APC-AF@750) |
| --- | --- | --- | --- | --- | --- | --- | --- | --- | --- | --- |
| Tube-1 | CD20 | CD123 | CD304 | CD86 | CD73 | CD34 | CD10 | CD19 | CD45 | CD38 |
| Clone | 2H7 | 9F5 | AD517F6 | HA5.2B7 | AD2 | 8G12 | ALB1 | J3-119 | J.33 | LS198-4-3 |
| Company | BL | BD | MB | BC | BD | BD | BC | BC | BC | BC |
| Tube-2 | HLA-DR | CD117 | CD15 | CD13 | CD19 | CD34 | CD56 | CD7 | CD45 | CD38 |
| Clone | L243 | YB5.B8 | 80H5 | SJ1D1 | J3-119 | 8G12 | N901 (HLDA6) | 8H8.1 | J.33 | LS198-4-3 |
| Company | BL | BD | BC | BC | BC | BD | BC | BC | BC | BC |
| Tube-3 | HLA-DR | CD36 | CD163 | CD123 | CD64 | CD33 | CD117 | CD34 | CD45 | CD38 |
| Clone | L243 | FA6.152 | GHI/61 | 9F5 | 22 | D3HL60.251 | 104D2D1 | 581 | J.33 | LS198-4-3 |
| Company | BL | PB_BC | BD | BC | BC | BC | BC | BC | BC | BC |
| Tube-4 | sCD3 | CD5 | CD4 | CD7 | CD34 | TCR γδ | CD2 | CD1a | CD45 | CD8 |
| Clone | OKT3 | UCHT2 | 13B8.2 | 8H8.1 | 581 | IMMU510 | 39C1.5 | BL6 | J.33 | B9.11 |
| Company | BL | BD | BC | BC | BC | BC | BC | BC | BC | BC |
| Tube-5 | x | CD117 | AMPO | cyto CD79a | cytoCD3 | x | CD22 | CD34 | CD45 | CD11b |
| Clone | x | YB5.B8 | CLB-MPO-1 | HM47 | UCHT1 | x | SJ10.1H11 | 581 | J.33 | Bear1 |
| Company | x | BD | BC | BC | BC | x | BC | BC | BC | BC |
| Abbreviations: BC, Beckman Coulter; BD, Becton Dickinson biosciences; BL, BioLegend; MB, Miltenyi Biotec, TF, Thermofisher | | | | | | | | | | |

**Supplementary Table S2. 10-color 11-antibody T-cell ALL MRD panel.**

| Fluorochromes | BV510 | BV421 | FITC | PE | ECD | PC5.5 | PC7 | APC | APC-A700 | APC-A750 |
| --- | --- | --- | --- | --- | --- | --- | --- | --- | --- | --- |
| Main MRD tube | CD16 & CD56 | CD3 | CD8 | CD7 | cytoCD3 | CD34 | CD5 | CD4 | CD45 | CD38 |
| Clone | 3G8 &  HCD56 | UCHT1 | B9.11 | 8H8.1 | UCHT1 | 8G12 | BL1a | 13B8.2 | J.33 | LS198-4-3 |
| Company | BL | BD | BC | BC | BC | BD | BC | BC | BC | BC |
| Syto13 tube | x | x | Syto13 | x | x | x | x | CD7 | CD45 | x |
| Clone |  |  |  |  |  |  |  | 8H8.1 | J.33 |  |
| Company |  |  | TF |  |  |  |  | BC | BC |  |
| Additional T-ALL MRD antibody panel | | | | | | | | | | |
| Fluorochromes | CD16+ CD56 | CD3 | Anti-TdT | CD7 | Cyto CD3 | CD13+ CD33 | CD117 | CD1a | CD45 | CD38 |
| Clone | 3G8/HCD56 | UCHT1 | HTDT-6 | 8H8.1 | UCHT1 | Immu103.44/D3HL60.251 | 104  D2D1 | BL6 | J.33 | LS198-4-3 |
| Company | BL | BL | Supratech | BC | BC | BC | BC | BC | BC | BC |
| Abbreviations: BC, Beckman Coulter; BD, Becton Dickinson biosciences; BL, BioLegend; TF, ThermoFisher Scientific (Invitrogen). | | | | | | | | | | |

**Supplementary Figure S1 – Diagnostic immunophenotype**

Supplementary Figure 1. Part A: Dot plots A1 to A9 demonstrates the immunophenotype in a diagnostic sample from a patient of T-cell acute lymphoblastic leukemia (T-ALL). The blast cells (red dots) demonstrated strong expression of CD7, intermediate expression of CD5 and dual expression of CD4/CD8 and cytoplasmic CD3 but are negative for surface CD3, CD22 and CD56. CD34 and CD1a are positive in the minute subsets of blast population (dot plot A8).


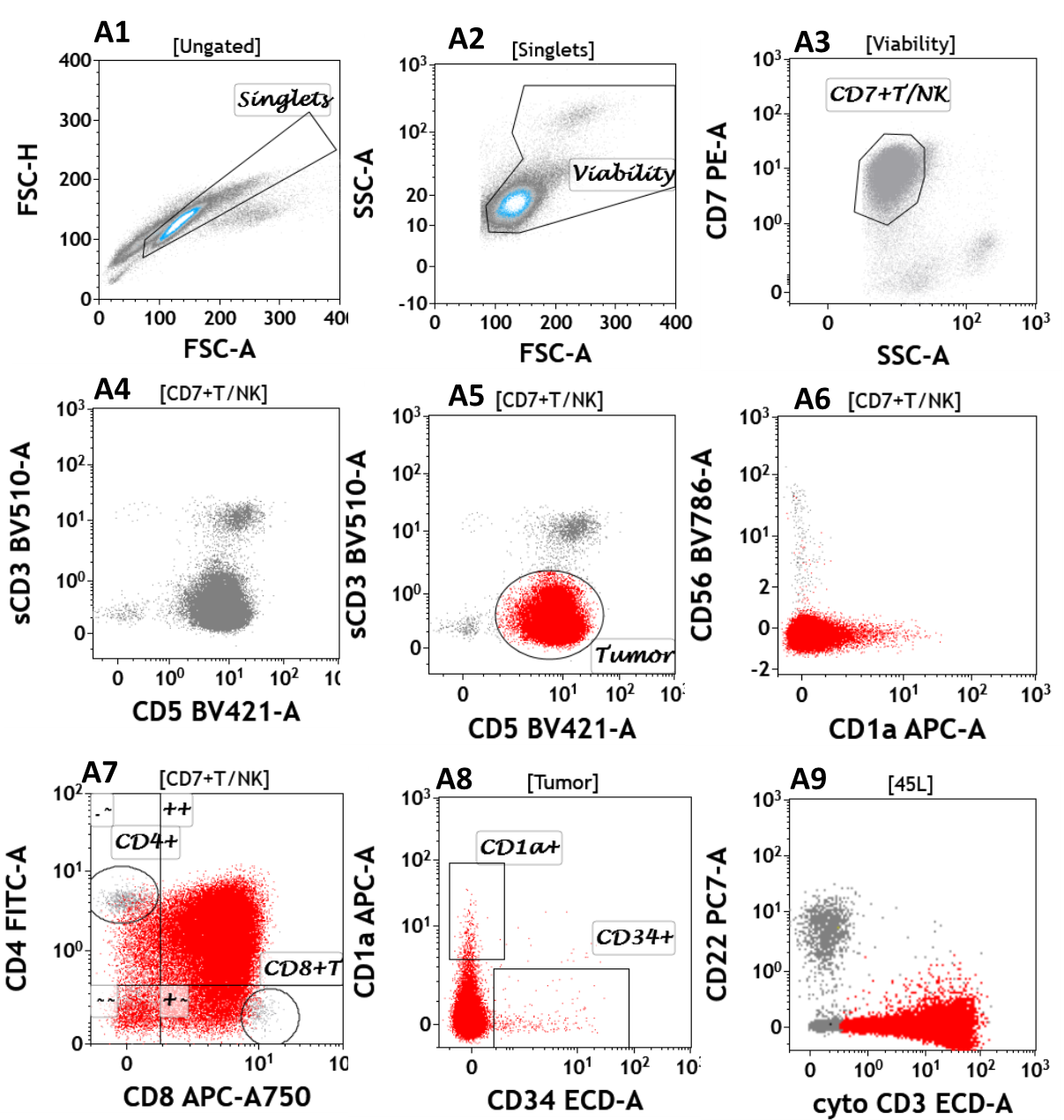


**Supplementary Figure S2 – MRD analysis**

Dot plots B1 to B8 demonstrated an approach of T-ALL MRD analysis in the post-induction (PI-MRD) bone marrow sample. The blast cells (red dots) demonstrated antigen expression similar to its diagnostic immunophenotype (shown in part-A). Residual blasts are positive for CD7, CD5 and dual expression of CD4/CD8 and cytoplasmic CD3 but are negative for surface CD3 and CD56. CD34 is positive in a minute subset of the blast population (dot plot B8).

**B1**

**B2**

**B3**

**B4**

**B5**

**B6**

**B7**

**B8**

Total No of viable events = 4 119 552.

MRD events = 1 148.

**MRD = 0.028%**

| **Supplementary Table S3. Hazard ratios (95% CI) of four risk groups using PI-MRD status and hyperleukocytosis at diagnosis with RFS, EFS and OS as the outcome (n=256).** | | | | |
| --- | --- | --- | --- | --- |
| Risk Groups | **0** | **1** | **2** | **3** |
| RFS  (*p*< 0.0001) | | | | |
| 0 | - | 3.5 (1.89 to 6.48) | 6.45 (3.44 to 12.1) | 13.96 (6.87 to 28.4) |
| 1 | 0.29 (0.15 to 0.53) | - | 1.84 (0.93 to 3.64) | 3.4 (1.87 to 8.53) |
| 2 | 0.16 (0.08 to 0.29) | 0.54 (0.27 to 1.07) | - | 2.17 (1.01 to 4.66) |
| 3 | 0.07 (0.035 to 0.15) | 0.25 (0.12 to 0.53) | 0.46 (0.21 to 0.99) | - |
| EFS  (*p*< 0.0001) | | | | |
| 0 | - | 2.56 (1.56 to 4.19) | 2.55 (1.54 to 4.22) | 4.84 (2.75 to 8.5) |
| 1 | 0.4 (0.24 to 0.64) | - | 1.0  (0.58 to 1.72) | 1.9 (1.04 to 3.44) |
| 2 | 0.4 (0.27 to 0.65) | 1.0 (0.58 to 1.73) | - | 1.9 (1.03 to 3.48) |
| 3 | 0.21 (0.12 to 0.36) | 0.53 (0.29 to 0.96) | 0.53 (0.29 to 0.99) | - |
| OS  (*p*= 0.0028) | | | | |
| 0 | - | 2.24 (1.24 to 4.04) | 1.88 (1.05 to 3.38) | 3.3 (1.73 to 6.30) |
| 1 | 0.45 (0.25 to 0.81) | - | 0.84 (0.45 to 1.59) | 1.47 (0.74 to 2.94) |
| 2 | 0.53 (0.29 to 0.95) | 1.19 (0.63 to 2.24) | - | 1.75 (0.88 to 3.49) |
| 3 | 0.30 (0.16 to 0.58) | 0.68 (0.34 to 1.36) | 0.57 (0.29 to 1.14) | - |
|  |  |  |  |  |


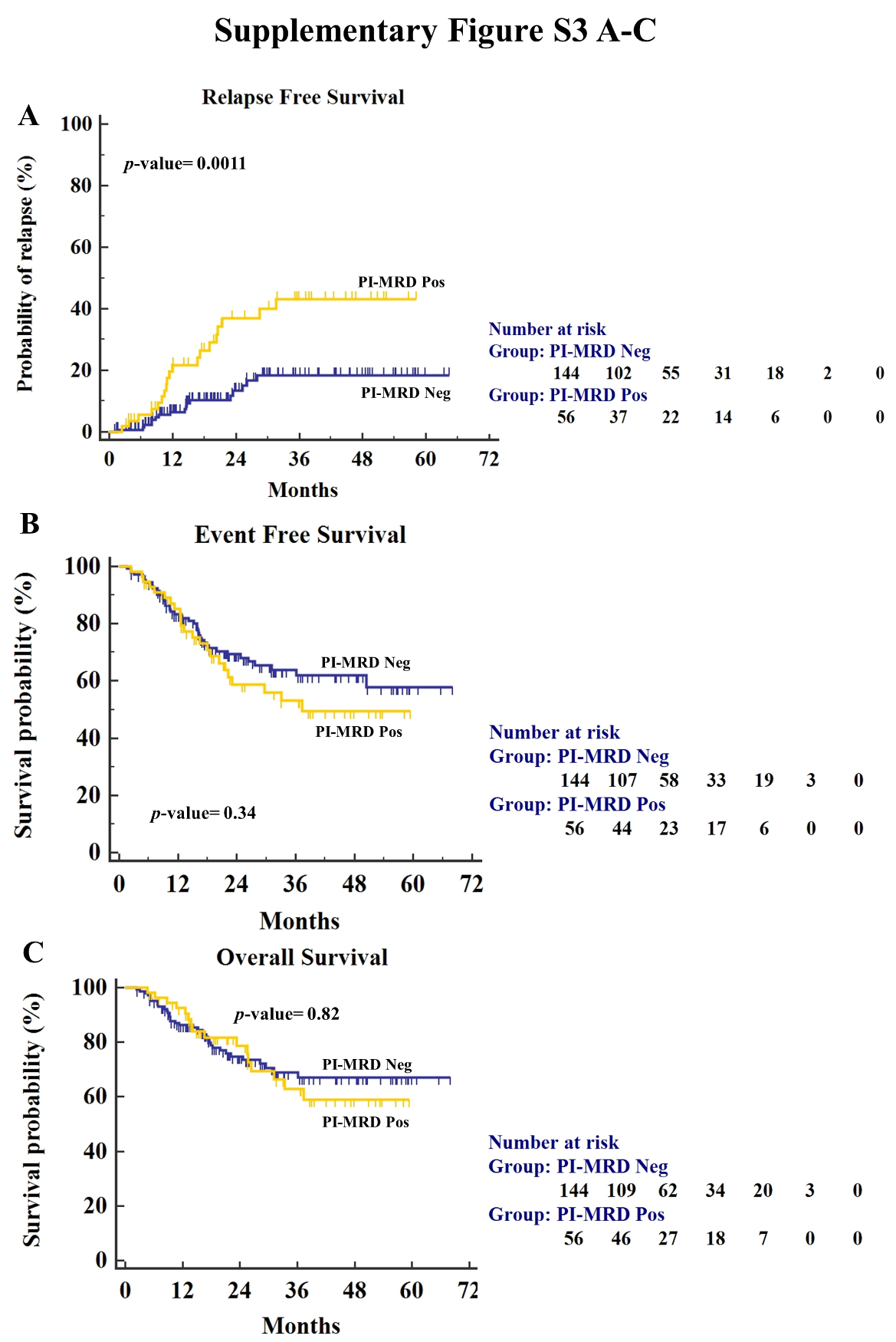


**Supplementary Figure S3 (A-C).** (A) Relapse-free survival (RFS), (B) Event-free survival (EFS) and (C) Overall Survival (OS) for patients stratified by PI-MRD negative versus PI-MRD positive but PC-MRD negative status (Kaplan–Meier analysis).
